# Normal thyroid stimulating hormone is associated with all-cause mortality in patients with acute myocardial infarction after percutaneous coronary intervention

**DOI:** 10.1186/s40001-023-01149-9

**Published:** 2023-06-29

**Authors:** Wei-cheng Ni, Shu-ting Kong, Ken Lin, Yu-heng Huang, Jun-feng Li, San-ling Shi, Yu-cheng Lu, Ling Cheng, Chang-xi Chen, Hao Zhou

**Affiliations:** 1 Department of Cardiology, The First Affiliated Hospital of Wenzhou Medical University, Wenzhou, China; 2 Department of Cardiology, Jin Hua Municipal Central Hospital, Jinhua, China

**Keywords:** Acute myocardial infarction, Global Registry of Acute Coronary Events score, Mortality, Percutaneous coronary intervention, Thyroid stimulating hormone

## Abstract

**Background:**

Circulating thyroid-stimulating hormone (TSH) levels within the normal reference range can affect the cardiovascular system. The present study investigated the prognostic value of normal TSH levels in patients presenting with acute myocardial infarction (AMI) following percutaneous coronary intervention (PCI).

**Methods:**

Between January 2013 and July 2019, 1240 patients with AMI and normal thyroid function were enrolled and classified according to TSH tertile. The trial endpoint was all-cause mortality. The integrated discrimination index (IDI) and the net reclassification index (NRI) were used to assess the combined predictive values of the TSH levels and the Global Registry of Acute Coronary Events (GRACE) scores.

**Results:**

After a median 44.25-month follow-up, 195 individuals died. Even after covariate adjustment by multivariate Cox regression (HR: 1.56; 95% CI 1.08–2.25; P = 0.017), the patients in the third TSH tertile were at the highest risk of all-cause mortality. A subgroup analysis revealed significant interactions between the TSH levels and the GRACE scores (high risk vs. low/medium risk) (P = 0.019). The addition of the TSH levels to the GRACE scores substantially improved the prediction of all-cause mortality, especially for high-risk patients (NRI = 0.239; IDI = 0.044; C-statistic value range 0.649–0.691; all significant).

**Conclusions:**

The third TSH tertile is associated with a higher incidence of all-cause mortality than the first TSH tertile in high-risk patients presenting with AMI after PCI.

## Introduction

There is a considerable risk of cardiovascular events in patients with acute myocardial infarction (AMI) despite substantial progress in percutaneous coronary intervention (PCI) and antithrombotic medication [1, 2]. Characterization of the factors contributing to this residual risk may reveal new strategies to mitigate it.

Slight fluctuations in thyroid-stimulating hormone (TSH) levels significantly affect the cardiovascular system [3]. Patients with subclinical hypothyroidism or hyperthyroidism may be at a substantially higher risk of cardiovascular death and cardiac events than healthy individuals [4, 5]. Reference-range TSH levels are positively correlated with the development of hypothyroidism [6]. For these reasons, certain medical specialists have recommended lowering the upper TSH reference level [7]. High reference-range TSH levels are associated with hypertension [8], unhealthy body mass index (BMI), abnormal blood lipid metabolism [9], reduced glomerular filtration [10], recurrence of atrial tachyarrhythmia after catheter ablation of atrial fibrillation [11], cardiovascular disease (CVD), and all-cause mortality in patients with diabetes [12]. Prior research demonstrated that even within the reference range, the third TSH tertile is correlated with mortality in patients with coronary artery disease (CAD) who have undergone PCI [24].

To the best of our knowledge, few studies have investigated the prognostic value of normal TSH levels in patients with AMI [13]. The present study aimed to assess the value of reference-range TSH levels in the prognosis of all-cause mortality in a relatively large cohort of patients presenting with AMI who have undergone PCI.

## Methods

### Study population

**Fig. 1:**
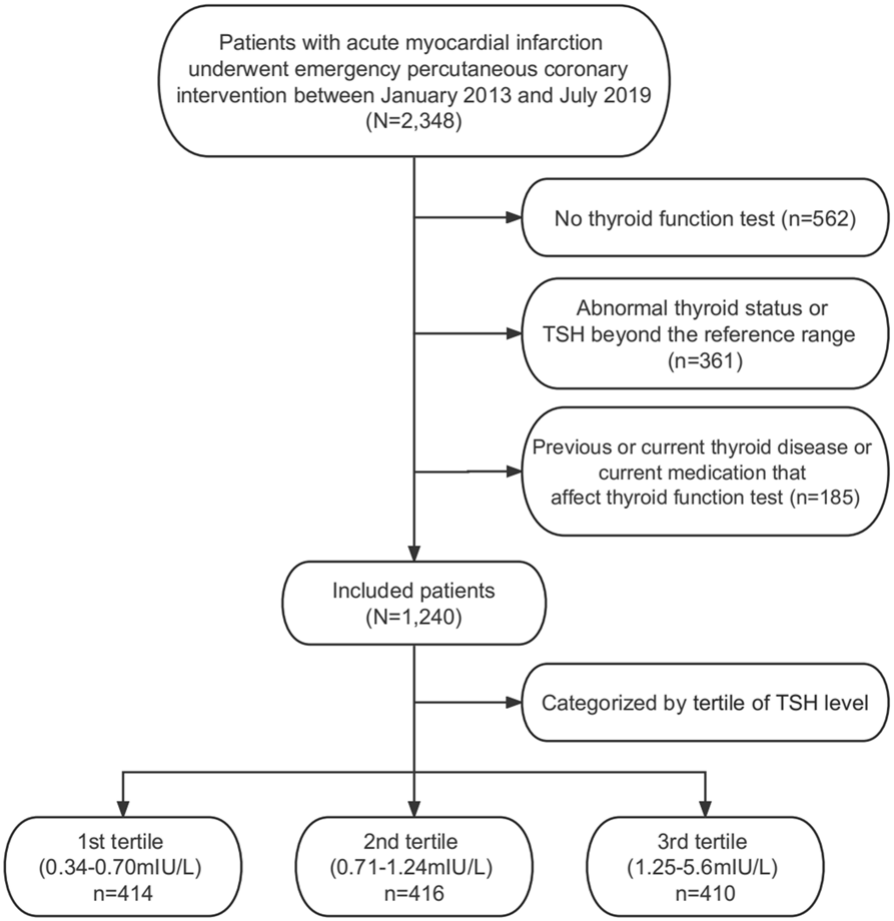
Flow chart of the study

This retrospective observational cohort analysis consisted of 2348 patients who presented with AMI undergoing percutaneous coronary intervention (PCI) at the First Affiliated Hospital of Wenzhou Medical University between January 2013 and July 2019. Details of the recruitment process are shown in Fig. 1. The exclusion criteria were as follows: (1) missing thyroid function test (n = 562); (2) previous or current thyroid disease or current medication that could affect thyroid function, including thyroid hormone, antithyroid drugs, lithium, steroids, and amiodarone (n = 185); and (3) TSH levels beyond the 0.34–5.60 mIU/L reference range, or abnormal thyroid status (n = 361). A total of 1240 participants with normal thyroid function were enrolled in the present study, and the Ethics Review Board of the First Affiliated Hospital of Wenzhou Medical University approved the trial protocol.

### Percutaneous coronary intervention (PCI) and medication

The pre-PCI treatment consisted of aspirin, clopidogrel, or ticagrelor administration. Contraindications were excluded, patients received the glycoprotein IIb/IIIa inhibitor tirofiban and supplemental heparin, and dosages were based on body weight. The activated clotting time target range was 250–300 s during the procedure. After the PCI, aspirin, clopidogrel, or ticagrelor was administered as part of a standard 12-mo dual antiplatelet regimen. Beta-blockers, statins, angiotensin-converting enzyme inhibitors (ACEIs), and angiotensin II receptor blockers (ARBs) were administered according to recommended treatment protocols [1, 2].

### Clinical laboratory data collection

Clinical, laboratory, medication-at-discharge, and procedural baseline data were collected and the laboratory results were recorded at admission. Within 24 h of admission, patients fasted for > 8 h, and samples of their venous blood were collected and immediately analyzed in preparation for the thyroid function test and other routine biochemical measurements. The thyroid function test measured the TSH, triiodothyronine (T3), thyroxine (T4), free triiodothyronine (FT3), and free thyroxine (FT4) levels, and their reference ranges were 0.34–5.6 mIU/L, 1.34–2.73 nmol/L, 78.38–157.4 nmol/L, 3.28–6.47 pmol/L, and 7.64–16.03 pmol/L, respectively. Patients that were not being administered levothyroxine or antithyroid medications and had TSH, FT3, and FT4 within their respective reference ranges were considered to be euthyroid or have normal thyroid function.

### Outcomes and follow-up

The target outcome was all-cause mortality. Every six months after the PCI, outcome data were collected using medical records, outpatient clinician visits, and telephone conversations.

### Statistical analysis

Categorical variables were presented as frequencies and percentages. Continuous variables were reported as medians and interquartile ranges when the distributions were skewed. Means and standard deviations were reported when the distributions were normal. Continuous variables were compared using analysis of variance (ANOVA) or the Kruskal–Wallis test. Categorical variables were compared by Pearson’s χ^2^ or Fisher’s exact test.

Kaplan–Meier curves were plotted to visualize temporal mortality distributions for patients in different TSH tertiles. All patient groups were compared by the log-rank test. Statistical adjustments accounted for age, sex, hypertension, diabetes, smoking history, hyperlipidemia, chronic kidney disease, previous coronary heart disease, Killip class, left ventricular ejection fraction (LVEF), GRACE score, three-vessel disease, and medications at discharge (beta-blockers, ACEI/ARB, and statins). Factors such as medications at discharge were extracted from Table 1 if they were clinically meaningful and strongly correlated with the risk of all-cause mortality. Parameters that significantly differed among the patients in various TSH tertiles were also considered. Variables such as age and sex linked to the TSH levels and the risk of adverse events were regarded as potential confounders. The final model was stabilized by removing highly collinear variables. The variables extracted from Table 1 were integrated into a forward-selected adjusted model. Covariates with variance inflation factor (VIF) ≥ 10 were removed from the final model. Hazard ratios (HR) were presented along with their corresponding 95% confidence intervals (CI).

**Table 1:** Baseline patient characteristics

Variables	1st tertile (n = 414)	2nd tertile (n = 416)	3rd tertile (n = 410)	P-value
Clinical characteristics				
Age	65.23 ± 11.74	68 ± 12.32	70.31 ± 12.71	< 0.001
Male, n (%)	371 (89.61%)	332 (79.81%)	300 (73.17%)	< 0.001
Diabetes mellitus, *n* (%)	86 (20.77%)	77 (18.51%)	101 (24.63%)	0.094
Hypertension, n (%)	246 (59.42%)	235 (56.49%)	225 (54.88%)	0.41
Smoking history, n (%)	235 (56.76%)	232 (55.77%)	227 (55.37%)	0.917
Hyperlipidaemia, n (%)	277 (66.91%)	268 (64.42%)	254 (61.95%)	0.331
Chronic kidney disease, n (%)	34 (8.21%)	39 (9.38%)	70 (17.07%)	< 0.001
Previous coronary heart disease, n (%)	23 (5.56%)	14 (3.37%)	20 (4.88%)	0.304
Peripheral arterial disease, n (%)	78 (18.84%)	87 (20.91%)	81 (19.76%)	0.755
Killip class ≥ II, n (%)	72 (17.39%)	88 (21.15%)	97 (23.66%)	0.082
LVEF ≤ 40%, n (%)	66 (15.94%)	56 (13.46%)	65 (15.85%)	0.527
Diagnosis				0.059
STEMI	353 (85.27%)	361 (86.78%)	332 (80.98%)	
NSTEMI	61 (14.73%)	55 (13.22%)	78 (19.02%)	
GRACE scores, median (Q1, Q3)	112.5 (97,128)	117 (98,134)	121 (102,142)	< 0.001
High risk status	177 (42.75%)	203 (48.8%)	210 (51.22%)	0.043
Laboratory characteristics				
White blood cells, median (Q1, Q3)	11.96 (9.81,14.38)	10.68 (8.84,13.31)	10.45 (8.62,13.33)	< 0.001
Haemoglobin (g/L), median (Q1, Q3)	136 (125.25,145)	133 (120.75,145)	129 (117,140)	< 0.001
Platelet (g/L), median (Q1, Q3)	213.5 (182,257)	212.5 (179,252)	210 (172,253)	0.458
Total cholesterol (mmol/L), median (Q1, Q3)	4.94 (4.31,5.66)	4.9 (4.22,5.74)	4.77 (4.08,5.59)	0.185
LDL-C (mmol/L), median (Q1, Q3)	3.13 (2.52,3.68)	3.1 (2.49,3.7)	2.96 (2.41,3.55)	0.112
Troponin(ng/ml), median (Q1, Q3)	16.47 (1,50)	13.71 (1.45,50)	11.58 (1.85,42.97)	0.586
CK-MB(U/L), median (Q1, Q3)	284.5 (151,503)	245 (130,437.5)	194 (91,397.75)	< 0.001
Creatinine(mg/dL), median (Q1, Q3)	67 (58.25,80)	68.5 (58,81.25)	69 (57,87)	0.438
Lactate(mmol/L), median (Q1, Q3)	2.7 (2.1,3.7)	2.7 (2.08,3.62)	2.8 (2.2,3.8)	0.094
TSH(mIU/L), median (Q1, Q3)	0.53 (0.45,0.62)	0.92 (0.8,1.05)	1.83 (1.47,2.5)	< 0.001
Triiodothyronine(nmol/L), median (Q1, Q3)	1.1 (0.93,1.32)	1.12 (0.95,1.32)	1.16 (0.97,1.33)	0.066
Thyroxine(nmol/L), median (Q1, Q3)	99.13 (86.45,114.52)	99.57 (85.23,113.88)	100.1 (86.86,114.64)	0.602
Free triiodothyronine (pmol/L), median (Q1, Q3)	4.2 (3.8,4.7)	4.24 (3.9,4.8)	4.3 (3.9,4.7)	0.543
Free thyroxine(pmol/L), median (Q1, Q3)	10.98 (9.81,12.52)	10.95 (9.86,12.5)	11.23 (9.99,12.7)	0.232
Medications at discharge				
Aspirin, n (%)	413 (99.76%)	416 (100%)	409 (99.76%)	0.554
Clopidogrel, n (%)	412 (99.52%)	415 (99.76%)	409 (99.76%)	0.78
Beta-Blockers, n (%)	347 (83.82%)	341 (81.97%)	306 (74.63%)	0.002
ACEI/ARBs, n (%)	251 (60.63%)	267 (64.18%)	236 (57.56%)	0.149
Statins, n (%)	402 (97.1%)	410 (98.56%)	396 (96.59%)	0.179
Procedural characteristics				
LM stenosis ≥ 50, n (%)	9 (2.17%)	14 (3.37%)	14 (3.41%)	0.494
LAD stenosis ≥ 50, n (%)	324 (78.26%)	339 (81.49%)	315 (76.83%)	0.243
LCA stenosis ≥ 50, n (%)	174 (42.03%)	190 (45.67%)	177 (43.17%)	0.556
RCA stenosis ≥ 50, n (%)	218 (52.66%)	220 (52.88%)	231 (56.34%)	0.493
Three-vessel disease, n (%)	94 (22.71%)	110 (26.44%)	116 (28.29%)	0.175
Calcification, n (%)	28 (6.76%)	41 (9.86%)	39 (9.51%)	0.224
Thrombus, n (%)	189 (45.65%)	177 (42.55%)	156 (38.05%)	0.085

STEMI, ST-segment elevation myocardial infarction; NSTEMI, Non-ST-segment elevation myocardial infarction; GRACE, Global Registry of Acute Coronary Events; LVEF, left ventricular ejection fraction; LDL-C, low-density lipoprotein cholesterol level; CK-MB-creatine kinase isoenzymes; TSH, thyroid stimulating hormone; ACEI/ARB, angiotensin converting enzyme inhibitor/angiotensin receptor blocker; LM- left main disease; LAD, left anterior descending coronary; LCX, left circumflex artery; RCA, right coronary artery

A restricted cubic spline analysis including the 25th, 50th, and 75th percentiles was performed on the nonlinear association between the TSH concentration and the all-cause mortality within the first and 95th percentiles to minimize the influence of potential outliers. The 25th percentile was the reference, and nonlinearity was identified by the likelihood ratio test.

The TSH level was combined with the GRACE score to evaluate the discrimination and reclassification performance of TSH. The GRACE score has been extensively used to predict mortality in patients with AMI [14]. The C-statistic, continuous net reclassification improvement (NRI), and integrated discrimination improvement (IDI) were also calculated. Statistical significance was defined as two-tailed P < 0.05. R v. 4.0.3 (R Core Team, Vienna, Austria) and SPSS v. 25.0 (IBM Corp., Armonk, NY, USA) were used for all statistical analyses.

## Results

### Baseline characteristics

Out of the 2348 consecutive patients presenting with AMI and undergoing emergency PCI during the study period, 1108 were excluded (Fig. 1). The remaining 1,240 patients had a mean age of 67.8 ± 12.4 yrs and 80.9% of them were male. Of these, 706 (56.9%) had hypertension, 264 (21.3%) were previously diagnosed with diabetes, and 799 (64.4%) had hyperlipidemia. Table 1 lists the baseline clinical, laboratory, medication, and procedural data of the study groups. The patients in the third TSH tertile were significantly older and there were fewer males than those in the other two groups. The prevalence of chronic kidney disease and the number of high-risk patients (according to the GRACE score) were significantly higher in the third TSH tertile than in the other two groups. The white blood cell (WBC) counts, the hemoglobin and creatine kinase isoenzyme (CK-MB) levels, and the rates of beta-blocker consumption were significantly lower in the patients of the third TSH tertile than they were in those of the other two groups.

### Association between TSH level and all-cause mortality

**Fig. 2:**
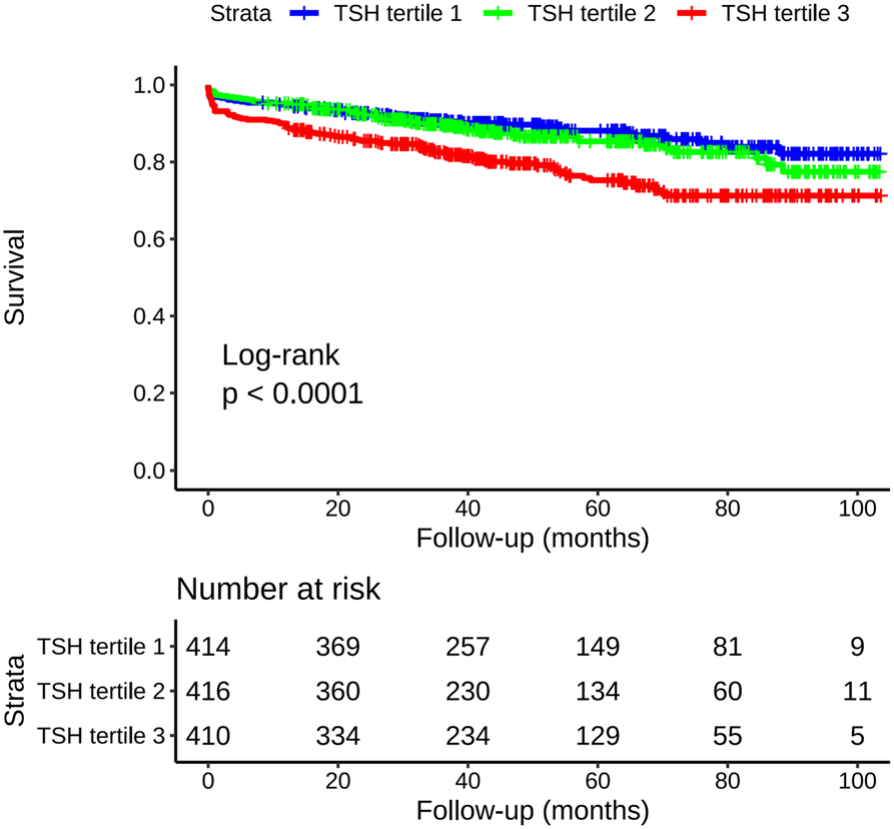
Kaplan–Meier analysis for all-cause mortality according to tertiles of TSH level

The median and interquartile ranges of the follow-up times were 44.25 mo and 30.64–67.23 mo, respectively. All-cause mortality occurred in 195 (15.7%) patients. There were 49 (11.8%), 56 (13.4%), and 90 (21.9%) deaths in the first, second, and third TSH tertile, respectively. The Kaplan–Meier plot demonstrated that all-cause death at follow-up was significantly higher for the patients in the third tertile than it was for those in the first and second tertile (log-rank test P < 0.001; Fig. 2).

The univariate analysis in Table 2 shows that the patients in the third TSH tertile were at a higher relative risk of all-cause mortality (HR = 2.01; 95% CI 1.42–2.85) than those in the first but not those in the second TSH tertile (HR: 1.19; 95% CI 0.81–1.74). Covariate adjustment by multivariate Cox regression disclosed that the association between the third TSH tertile and all-cause mortality lasted longer (HR: 1.56; 95% CI 1.08–2.25; P = 0.017) than that between the first TSH tertile and all-cause mortality. Age > 65 yrs, diabetes mellitus, chronic kidney disease, Killip class ≥ II, LVEF ≤ 40%, ACEI/ARB administration at discharge, three-vessel disease, and high-risk status were independently associated with all-cause death.

**Table 2:** Results of univariate and multivariate Cox proportional hazards model applied to assess predictors of all-cause mortality

Variables	Univariate analysis HR[95%CI]	P-value	Multivariate analysis HR[95%CI]	P-value
TSH, categorical				
1st	Ref.	–	Ref.	–
2nd	1.19[0.81,1.74]	0.384	1.11[0.75,1.65]	0.605
3rd	2.01[1.42,2.85]	< 0.001	1.56[1.08,2.25]	0.017
Age > 65 years	3.64[2.46,5.37]	< 0.001	1.87[1.15,3.02]	0.011
Male	0.59[0.43,0.8]	0.001	1.04[0.72,1.50]	0.822
Hypertension	1.53[1.14,2.06]	0.005	1.21[0.87,1.67]	0.259
Diabetes mellitus	1.76[1.3,2.38]	< 0.001	1.51[1.09,2.10]	0.013
Smoking history	1.01[0.76,1.35]	0.918	1.23[0.91,1.66]	0.178
Hyperlipidaemia	0.85[0.64,1.14]	0.278	0.93[0.69,1.25]	0.638
Chronic kidney disease	4.86[3.59,6.58]	< 0.001	2.09[1.46,2.98]	< 0.001
Previous coronary heart disease	2.01[1.22,3.31]	0.006	1.10[0.65,1.85]	0.718
Killip class ≥ II	3.39[2.55,4.5]	< 0.001	2.05[1.50,2.82]	< 0.001
LVEF ≤ 40%	3.07[2.27,4.14]	< 0.001	2.28[1.67,3.12]	< 0.001
Beta-Blockers	0.56[0.42,0.77]	< 0.001	0.88[0.63,1.22]	0.443
ACEI/ARBs	0.46[0.35,0.61]	< 0.001	0.52[0.38,0.70]	< 0.001
Statins	0.66[0.31,1.41]	< 0.001	0.99[0.45,2.18]	0.981
Three-vessel disease	1.91[1.43,2.55]	< 0.001	1.47[1.09,2.00]	0.012
High risk status	3.36[2.45,4.62]	< 0.001	1.52[1.02,2.26]	0.04

TSH, thyroid stimulating hormone; LVEF, left ventricular ejection fraction; ACEI/ARB, angiotensin converting enzyme inhibitor/angiotensin receptor blocker; HR, hazard ratio; CI, confidence interval

**Fig. 3:**
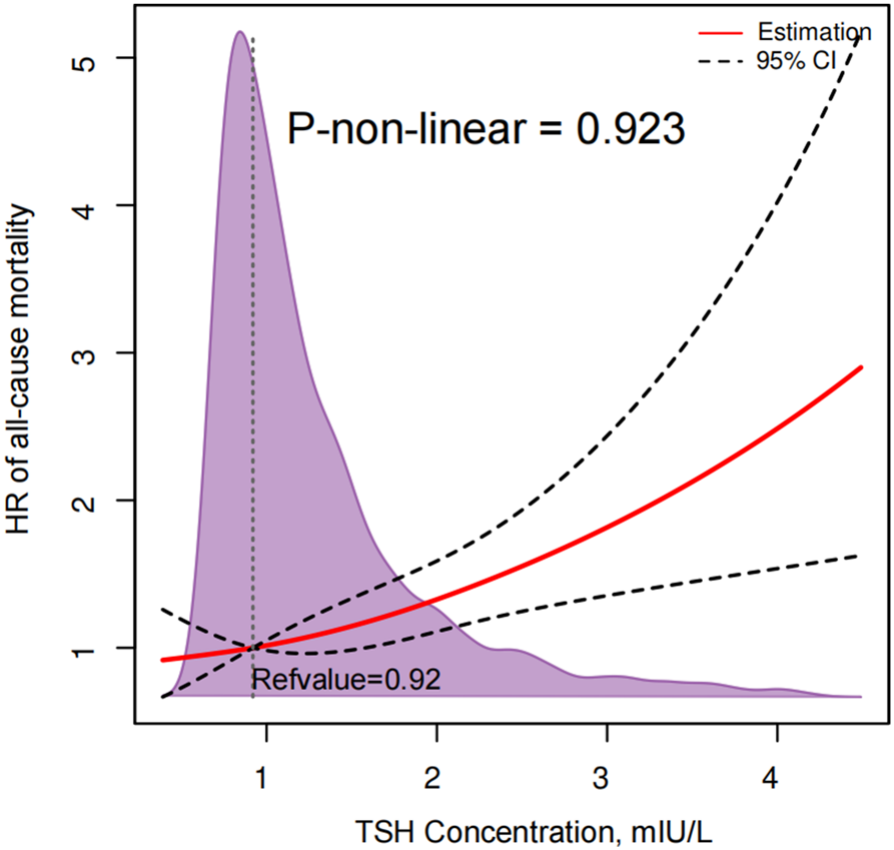
Restricted cubic splines of TSH concentration for hazard ratios of all-cause mortality in AMI patients. Red lines represent the hazard ratio, black dashed lines represent the 95% confidence intervals

The restricted cubic spline in Fig. 3 depicts the association between the TSH level and all-cause mortality after covariate adjustment by multivariate Cox regression. TSH levels > 0.92 mIU/mL were positively and linearly correlated with an increased risk of all-cause mortality (P_nonlinearity_ = 0.923).

### Subgroup analysis

Interactions among age, sex, diabetes, hypertension, clinical presentation, and GRACE score strata were evaluated after covariate adjustment by multivariate Cox regression. Strata variables were not included when they were stratified alone. Table 3 shows that the patients in the third TSH tertile were at higher risk of all-cause mortality than those in the first TSH tertile and at all strata analyzed except females, younger patients, and those with diabetes, non-ST-segment elevation myocardial infarction (NSTEMI), low/medium risk status, and no hypertension.

**Table 3:** Subgroup analysis on all-cause mortality across tertiles of TSH levels

TSH tertiles	No. of patients with events (%)			Adjusted HR [95% CI]			P for interaction
	1st tertile	2nd tertile	3rd tertile	1st tertile	2nd tertile	3rd tertile	
Total	49(11.8)	56(13.4)	90(21.9)	Ref.	1.11[0.75,1.65]	1.56[1.08,2.25]	
Age							0.34
> 65 years	38(17.1)	50(19.3)	77(28.3)	Ref.	1.18[0.76,1.83]	1.53[1,02,2.30]	
≤ 65 years	11(5.7)	6(3.7)	13(9.4)	Ref.	0.62[0.21,1.83]	1.40[0.53,3.70]	
Sex							0.555
Male	39(10.5)	43(12.9)	59(19.6)	Ref.	1.24[0.80,1.94]	1.59[1.04,2.43]	
Female	10(23.2)	13(15.4)	31(28.1)	Ref.	0.68[0.27,1.68]	1.08[0.49,2.4]	
Diabetes							0.944
Yes	15(17.4)	15(19.4)	30(29.7)	Ref.	0.97[0.44,2.13]	1.23[0.6,2.55]	
No	34(10.3)	41(12.0)	60(19.4)	Ref.	1.29[0.81,2.07]	1.73[1.11,2.69]	
Hypertension							0.729
Yes	31(12.6)	39(16.5)	59(26.2)	Ref.	1.39[0.85,2.26]	1.82[1.15,2.87]	
No	18(10.7)	17(9.3)	31(16.7)	Ref.	0.62[0.32,1.24]	0.97[0.52,1.82]	
Clinical presentation							0.380
STEMI	41(11.6)	45(12.4)	76(22.8)	Ref.	1.06[0.69,1.64]	1.76[1.18,2.63]	
NSTEMI	8(13.1)	11(20.0)	14(17.9)	Ref.	1.41[0.51,3.93]	1.65[0.70,3.89]	
GRACE score							0.019
High risk	30(16.9)	38(18.7)	75(35.7)	Ref.	1.27[0.77,2.01]	2.02[1.29,3.16]	
Low/medium risk	19(8.0)	18(8.4)	15(7.5)	Ref.	0.93[0.47,1.83]	0.69[0.33,1.45]	

GRACE score- Global Registry of Acute Coronary Events score; TSH- thyroid stimulating hormone; HR- hazard ratio; CI- confidence interval

For all-cause mortality, there was a significant interaction between the GRACE score (high risk or low/medium risk) and the TSH level (P_interaction_ = 0.019). For the subgroup with high-risk status, the HR for all-cause mortality was 2.02 (95% CI 1.29–3.16) in the third TSH tertile relative to the first TSH tertile (reference) group. For the subgroup with low/medium-risk status, the HR for all-cause mortality was 0.69 (95% CI 0.33–1.45) in the third TSH tertile relative to the first TSH tertile (reference) group. There were no significant interactions between the TSH and any other strata variable associated with the risk of all-cause mortality.

### Prognostic value of combining the TSH with the GRACE score

The prognostic value of combining the TSH with the GRACE score to predict all-cause mortality in patients with AMI is shown in Table 4. Compared with the baseline GRACE score model, the addition of TSH significantly improved reclassification based on the NRI (0.172; 95% CI 0.057–0.265; P < 0.001), IDI (0.024; 95% CI 0.009–0.054; P < 0.001), and C-statistic (0.732–0.742; P < 0.001). For the high-risk group, the addition of the TSH to the GRACE score significantly improved reclassification based on the NRI (23.9% improvement; 0.239; 95% CI 0.097–0.339; P = 0.007) and IDI (4.4% improvement; 0.044; 95% CI 0.014–0.093; P < 0.001) and significantly increased the C-statistic from 0.649 (95% CI 0.601–0.697) to 0.691 (95% CI 0.644–0.737) (P < 0.001). For the low/medium-risk group, however, there were no statistically significant incremental effects with respect to the C-statistic (0.710–0.711; P = 0.607), NRI (3.7% improvement; P = 0.492), or IDI (0.1% improvement; P = 0.555).

**Table 4:** Evaluation of the predictive value of TSH combined with GRACE score for all-cause mortality

	NRI		IDI		C-Statistic	
	Index(95% CI)	P-value	Index(95% CI)	P-value	Index(95% CI)	P-value
Total population						
GRACE score	–	Ref.	–	Ref.	0.732(0.696,0.767)	Ref.
GRACE score + TSH	0.172(0.057,0.265)	< 0.001	0.024(0.009,0.054)	< 0.001	0.742(0.706,0.779)	< 0.001
High risk group						
GRACE score	–	Ref.	–	Ref.	0.649(0.601,0.697)	Ref.
GRACE score + TSH	0.239(0.097,0.339)	0.007	0.044(0.014,0.093)	< 0.001	0.691(0.644,0.737)	< 0.001
Low / medium risk group						
GRACE score	–	Ref.	–	Ref.	0.710(0.641,0.779)	Ref.
GRACE score + TSH	0.037(− 0.082,0.173)	0.492	0.001(− 0.001,0.031)	0.555	0.711(0.642,0.781)	0.607

GRACE score- Global Registry of Acute Coronary Events score; TSH- thyroid stimulating hormone; NRI- net-reclassification index; IDI- integrated discrimination improvement; CI- confidence interval

## Discussion

The present study indicated that within its reference range, the third TSH tertile was independently associated with the risk of all-cause mortality in patients presenting with AMI after PCI. Moreover, a subgroup analysis revealed an interaction between the TSH level and the GRACE score. Hence, a high normal TSH level had prognostic value for patients at high (but not low/medium) risk of all-cause mortality. The present work also showed that the addition of the TSH improved the ability of the GRACE score to predict patients with AMI who are at high risk of all-cause mortality.

The pathophysiological mechanisms linking TSH levels in the upper part of the reference range to an elevated risk of all-cause mortality are not clear. TSH caused extrathyroidal effects such as in vitro interleukin-6 (IL-6) induction and tumor necrosis factor (TNF) biosynthesis [15, 16] as well as in vivo upregulation of nitric oxide (NO) metabolites [17]. Earlier studies proposed that elevated TSH increases the risk of all-cause mortality in patients with AMI by increasing mitochondrial oxidative stress, the proinflammatory state, thrombogenicity, and endothelial dysfunction, and by decreasing heart function [15, 17–20]. Here, the TSH level and all-cause mortality were strongly positively correlated even after adjusting for age, sex, and other potential confounders. Hence, the pathophysiological mechanisms by which the TSH level predicts all-cause death in patients with AMI remain to be elucidated.

TSH levels in the upper limit of the normal range indicate the early stages of hypothyroidism [6]. This condition is associated with mortality and cardiovascular events in various cardiovascular diseases (CVDs) and different symptoms in the general population [21, 22]. In a population-based study, a mediation analysis associated a high risk of all-cause mortality with CVD. Patients with high normal TSH were at greater risk of all-cause death and CVD than those with mid- to normal TSH [22]. High normal TSH was also associated with an increased risk of ventricular arrhythmia in patients with dilated cardiomyopathy. Thus, it is essential to monitor TSH levels [23]. A Ndrepepa trial on 8010 post-PCI patients revealed that third-tertile TSH within the reference range was associated with an elevated risk of all-cause death within the first 30 days of PCI but not between 30 days and three years after it [24]. However, a meta-analysis disclosed no correlations between normal-range TSH levels and the risks of coronary heart disease events and mortality [25].

To the best of our knowledge, only a few studies have focused on normal TSH levels in patients with AMI. A small-sample study conducted in Turkey demonstrated that patients in the high TSH tertile with acute coronary heart disease were at a higher risk of short-term all-cause death than those in the low- to mid-TSH tertile [13]. Observational research on a cohort of 1203 patients with ST-segment elevation myocardial infarction (STEMI) demonstrated a correlation between normal TSH levels and mortality in patients who did not undergo emergency reperfusion therapy. However, the foregoing parameters were not correlated in STEMI patients who were administered this treatment [26]. Nevertheless, a study on 1186 individuals showed that normal high TSH values did not affect the prognosis of patients with STEMI [27]. The results of the preceding studies were inconsistent as they did not control for low T3 syndrome or other types of abnormal thyroid function status. Low T3 syndrome is associated with poor prognosis in patients with heart disease and may, therefore, influence the relationship between the TSH level and mortality [28]. To the best of our knowledge, the present work is the first to demonstrate an association between high TSH levels within the reference range and long-term all-cause mortality in patients presenting with AMI following PCI. This study also confirmed that the TSH can enhance the ability of the GRACE score to predict all-cause mortality accurately.

The GRACE score has been extensively used in clinical settings to determine the prognosis of patients with AMI [14, 31, 35]. Our subgroup analysis revealed significant interactions between normal TSH levels and the GRACE score (high- or low/medium-risk status). The risk of all-cause mortality was similar across all TSH levels in patients with low/medium-risk status. High normal TSH levels were associated with an elevated risk of all-cause mortality in patients with high-risk status. The addition of the TSH significantly enhanced the prognostic value of the GRACE score by comparing the C-statistics, IDI, and NRI, thereby improving the prediction of the risk of all-cause mortality in patients with high-risk (but not low/medium-risk) status. Mortality related to high normal TSH levels was relatively greater in high-risk than low/medium-risk patients. Dysregulated basal metabolism and slightly abnormal peripheral thyroid hormone metabolism are often associated with poor prognosis in critically ill patients [29, 30]. We hypothesized that high normal TSH levels in patients with elevated GRACE scores might indicate aberrant thyroid metabolism and, therefore, a relatively high risk of all-cause mortality.

There is a lack of consensus as to whether thyroid hormone replacement therapy should be administered to patients with AMI. A study in Greece showed that acute triiodothyronine treatment improved postischemic cardiac function in patients with MI [32]. Another investigation reported that levothyroxine treatment did not improve left ventricular function in patients with AMI and subclinical hypothyroidism [33]. Relative to untreated controls, levothyroxine therapy nonsignificantly (P_interaction_ = 0.44) improved baseline reduced LVEF in certain patients. Low LVEF and high prognostic scores reflect high disease severity and adverse events in patients with AMI [14, 34–36]. Thus, thyroid hormone replacement therapy might be beneficial for patients with high GRACE scores. Future research in this area should be conducted on large cohorts.

Prior studies reported conflicting optima for TSH reference ranges [37, 38]. Serum TSH levels indicate thyroid-pituitary feedback, but may not reliably reflect thyroid function in all organs [39]. Therefore, adverse cardiovascular effects could occur even at TSH levels that lie within the reference range. The TSH reference range must, therefore, be re-evaluated for each type of condition as well as AMI.

## Limitations

The present study had several limitations. First, the TSH levels were only calculated at baseline. Hence, dynamic changes that might have occurred during follow-up were overlooked. Second, the present investigation was a single-center observational study. Therefore, potential confounders might not have been fully adjusted. Third, thyroid autoantibodies were not measured. They should have been considered as they might explain the pathophysiological mechanism by which high normal TSH levels are associated with the risk of all-cause mortality in patients presenting with AMI after PCI.

## Conclusion

High TSH levels within the reference range were independently associated with a high risk of all-cause mortality in patients presenting with AMI following PCI. The addition of TSH improves the prognostic value of the GRACE score in the stratification of the risk of all-cause mortality in the aforementioned patients.

## Data Availability

The data supporting this article will be made available by the authors upon reasonable written request.

## Abbreviations

ACEI: Angiotensin-converting enzyme inhibitor
AMI: Acute myocardial infarction
ARB: Angiotensin II receptor blocker
CAD: Coronary artery disease
CI: Confidence interval
CK-MB: Creatine kinase isoenzymes
FT3: Free triiodothyronine
FT4: Free thyroxine
GRACE: Global Registry of Acute Coronary Events
HR: Hazard ratio
IDI: Integrated discrimination improvement
IL-6: Interleukin-6
LAD: Left anterior descending coronary
LCX: Left circumflex artery
LM: Left main disease
LVEF: Left ventricular ejection fraction
NRI: Net reclassification improvement
NSTEMI: Non-ST-segment elevation myocardial infarction
PCI: Percutaneous coronary intervention
RCA: Right coronary artery
STEMI: ST-segment elevation myocardial infarction
T3: Triiodothyronine
T4: Thyroxine
TNF: Tumor necrosis factor
TSH: Thyroid stimulating hormone
VIF: Variance inflation factor
